# When to Pull the Trigger: Conceptual Considerations for Approximating Head Acceleration Events Using Instrumented Mouthguards

**DOI:** 10.1007/s40279-024-02012-5

**Published:** 2024-03-09

**Authors:** James Tooby, Kevin Till, Andrew Gardner, Keith Stokes, Gregory Tierney, Daniel Weaving, Steve Rowson, Mazdak Ghajari, Carolyn Emery, Melanie Dawn Bussey, Ben Jones

**Affiliations:** 1 Carnegie Applied Rugby Research (CARR) Centre, Carnegie School of Sport, Leeds Beckett University, Leeds, UK; 2 Leeds Rhinos Rugby League Club, Leeds, UK; 3 Sydney School of Health Sciences, Faculty of Medicine and Health, The University of Sydney, Camperdown, NSW Australia; 4 Centre for Health and Injury and Illness Prevention in Sport, University of Bath, Bath, UK; 5 Medical Services, Rugby Football Union, Twickenham, UK; 6 Sport and Exercise Sciences Research Institute, School of Sport, Ulster University, Belfast, UK; 7 Biomedical Engineering and Mechanics, Virginia Tech, Blacksburg, VA USA; 8 Leeds Beckett University, Leeds, UK; 9 Dyson School of Design Engineering, Imperial College London, London, UK; 10 Sport Injury Prevention Research Centre, Faculty of Kinesiology, University of Calgary, Calgary, AB Canada; 11 Departments of Pediatrics and Community Health Sciences, Cumming School of Medicine, University of Calgary, Calgary, AB Canada; 12 School of Physical Education Sport and Exercise Sciences, University of Otago, Dunedin, New Zealand; 13 Division of Physiological Sciences, Department of Human Biology, Faculty of Health Sciences, University of Cape Town and Sports Science Institute of South Africa, Cape Town, South Africa; 14 School of Behavioural and Health Sciences, Faculty of Health Sciences, Australian Catholic University, Brisbane, QLD Australia; 15 Rugby Football League, England Performance Unit, Red Hall, Leeds, UK; 16 Premiership Rugby, London, UK

## Abstract

Head acceleration events (HAEs) are acceleration responses of the head following external short-duration collisions. The potential risk of brain injury from a single high-magnitude HAE or repeated occurrences makes them a significant concern in sport. Instrumented mouthguards (iMGs) can approximate HAEs. The distinction between sensor acceleration events, the iMG datum for approximating HAEs and HAEs themselves, which have been defined as the in vivo event, is made to highlight limitations of approximating HAEs using iMGs. This article explores the technical limitations of iMGs that constrain the approximation of HAEs and discusses important conceptual considerations for stakeholders interpreting iMG data. The approximation of HAEs by sensor acceleration events is constrained by false positives and false negatives. False positives occur when a sensor acceleration event is recorded despite no (in vivo) HAE occurring, while false negatives occur when a sensor acceleration event is not recorded after an (in vivo) HAE has occurred. Various mechanisms contribute to false positives and false negatives. Video verification and post-processing algorithms offer effective means for eradicating most false positives, but mitigation for false negatives is less comprehensive. Consequently, current iMG research is likely to underestimate HAE exposures, especially at lower magnitudes. Future research should aim to mitigate false negatives, while current iMG datasets should be interpreted with consideration for false negatives when inferring athlete HAE exposure.

## Key Points

**Table:** 

The ability of instrumented mouthguards to approximate head acceleration events accurately is constrained by technical limitations.
There are multiple mechanisms that contribute to false positives and false negatives.
Post-processing algorithms and video verification can virtually eradicate false positives, whereas there are less means for mitigating false negatives.
The presence of false negatives in instrumented mouthguard datasets leads to underestimations of approximated head acceleration event exposures.

## Introduction

Short-term, medium-term and long-term consequences of brain injury are a concern across sports. Head acceleration events (HAEs) are defined as events resulting in an acceleration response of the head caused by an external short-duration collision force applied directly to the head or indirectly via the body [1]. A single HAE can result in an acute brain injury (e.g. concussion) [2]. Repeated HAEs that do not result in concussion symptoms [3] are also of interest because of the potential association with negative effects on cognition and other physiological outcomes [4], and may be considered as alternative injury mechanisms in themselves [5–8]. Measuring and characterising HAEs are important for guiding initiatives to reduce brain injury across sports.

Wearable devices instrumented with inertial sensors (e.g. accelerometers, gyroscopes) have the capability of approximating HAEs by measuring head acceleration. Inertial sensors have been embedded in headbands [9, 10], helmets [11–14], skull caps [9], skin patches [14–16], mouthpieces [17] and mouthguards [18–20], demonstrating varying degrees of accuracy, e.g., skin-based and skull cap-based sensors are displaced up to 4 and 13 mm from the ear canal during a soccer header whereas mouthguard sensors are displaced by less than 1 mm [21]. A lack of coupling to the skull can lead to erroneous head impact counts and acceleration magnitudes [22]; thus, instrumented mouthguards (iMGs) have a high potential to accurately measure head kinematics[21].

**Fig. 1:**
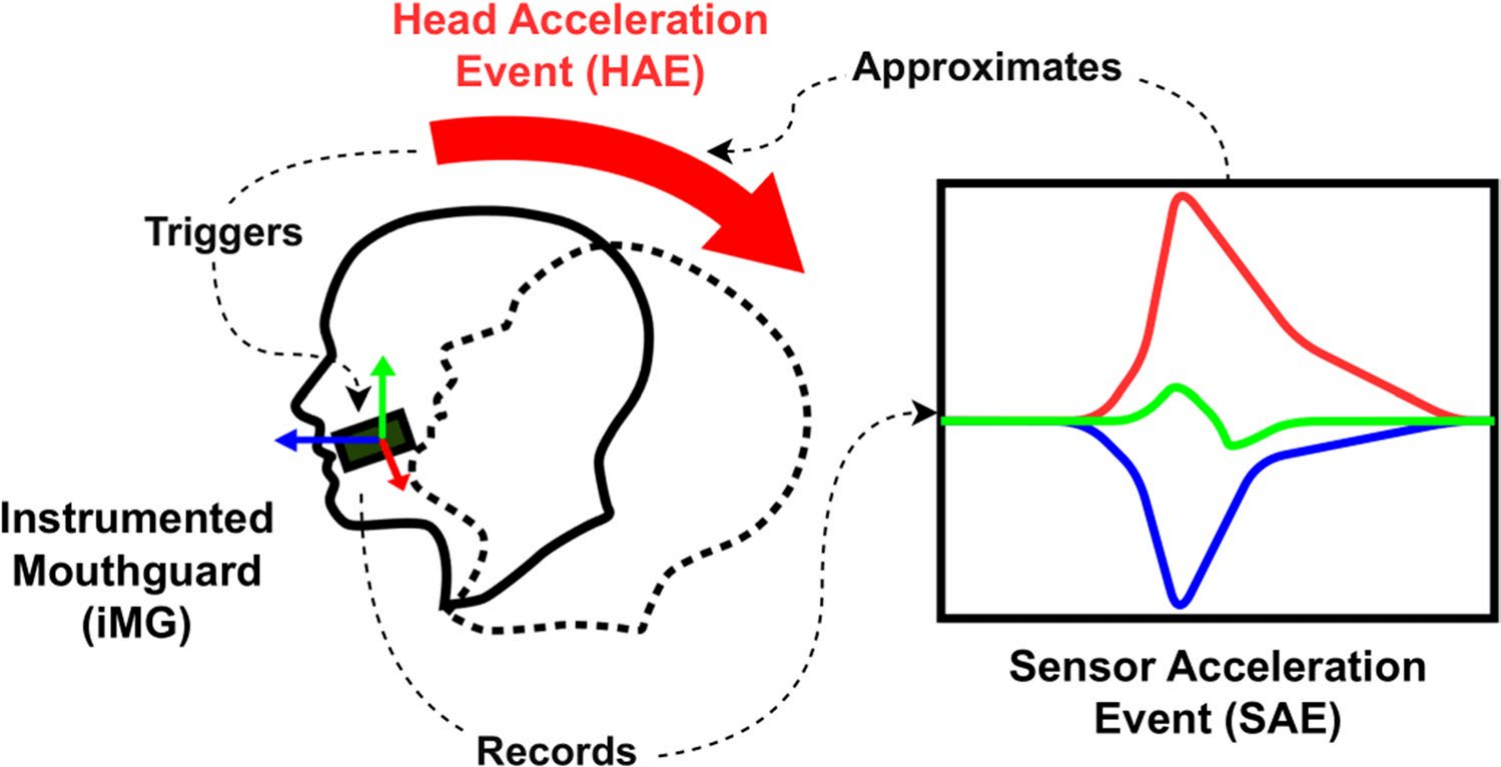
A sensor acceleration event (SAE) approximates a head acceleration event (HAE) when a head acceleration triggers an instrumented mouthguard (iMG) to record an SAE

The definition of an HAE is clearly outlined in the Consensus Head Acceleration Measurement Practices (CHAMP) [23] as the in vivo occurrence of head acceleration following a collision [1], irrespective of whether it is recorded. However, the term HAE is commonly used interchangeably with the iMG datum, a sensor acceleration event (SAE) [24]. An SAE contains sensor measurements that can be used to approximate the kinematics of HAEs (Fig. 1). The distinction between HAEs, the in-vivo event and iMG-recorded SAEs is made in the current article to discuss limitations associated with approximating HAEs using iMGs. For instance, SAEs can be recorded without an actual HAE occurring (i.e. false positives), or conversely, HAEs may occur without an SAE being recorded (i.e. false negatives) [1]. These errors can lead to misinterpretations of HAE exposure when using iMG data. Accordingly, this Current Opinion explores the technical constraints of iMGs for approximating HAEs and discusses the conceptual considerations for the interpretation of iMG data.

## Potential iMG Applications

The implementation of iMGs within sports presents the opportunity to understand and reduce brain injury across different settings. Prior to iMG application, sports have modified rules to reduce potential brain injury risk, e.g., removing the shoulder charge in rugby league [25], lowering the tackle height in rugby union [26] and disallowing body checks in youth ice hockey [27, 28]. By understanding the technical features of a sport that result in HAEs, governing bodies may use iMGs to inform rules and policy changes, and coaches or practitioners may use them to guide contact load management and technique education. Critically, iMGs have the capability of approximating HAE exposure, enabling their use for guiding and evaluating initiatives aimed at reducing HAE exposure in sport.

At a team level, iMGs can be used to identify training activities associated with the greatest risk of HAEs to determine whether these are deemed essential from performance and player welfare perspectives [29–32]. Likewise, it is possible to monitor HAE exposure on a player-by-player basis and implement contact load management strategies to reduce unnecessary exposure. Given the energetic and physiological cost of collisions [33–35], periodising contact load may improve performance by balancing the minimal dose needed to condition players to contact whilst reducing HAE exposure.

At a clinical level, iMGs may have clinical applications for assisting clinicians with the diagnosis of brain injury. Injury tolerance thresholds for concussion using wearable sensors have been elusive [36, 37] owing to the numerous intrinsic (e.g. head size [38], age [39], previous concussion history [40, 41]) and extrinsic (e.g. head protection [42]) factors that confound the clinical response to a given HAE [43]. As such, diagnosing concussion solely based on iMG data is not recommended. Furthermore, the diagnosis of concussion should always be a medical decision made by a clinician. Despite this, iMGs may support clinical decision making by alerting clinicians to high-magnitude SAEs associated with an elevated risk of concussion [2]; subsequent clinical diagnoses can be made using a range of available information (e.g. clinical assessment [44], video footage).

Head kinematics of HAEs provided by iMGs can also be used to understand the mechanism of brain injuries. Field data collected using iMGs can approximate HAE exposures and magnitudes to inform laboratory-based designs for investigating the biomechanical mechanisms at the cell structure level [45–47] and brain biomechanical model simulations [48]. Understanding the biomechanical mechanisms of brain injury is important to establish which HAEs are most likely to lead to brain injury, as well as informing the design of personal protective equipment [49] for reducing brain injury incidence within and beyond sport.

## Head Acceleration Magnitude

Appropriate interpretation of iMG data is necessary to realise the potential applications of iMGs. Specifically, which HAEs are important to iMG applications or research questions should be identified for interpreting iMG data. From injury prevention and player welfare perspectives, HAEs that have the potential to be clinically significant may be of interest. However, this magnitude has yet to be determined.

Injury risk curves suggest that there is a 50% chance of concussion from a 63 [50] to 81 *g* [51] HAE, based on helmeted SAEs in American Football using helmet-based sensors. However, it is worth noting that a diagnosed concussion has been reported with an iMG-recorded SAE as low as 53 *g* [52]. Consequently, if iMGs are being used solely for concussion prediction, then focusing on HAE magnitudes near to this may be appropriate. Despite this, HAEs that do not result in a diagnosed concussion (i.e. subconcussive HAEs) may still present a clinical effect. Retrospective research of retired American Football players has demonstrated that repetitive exposure to HAEs above 10–15 *g* is a better predictor than a concussion history of chronic traumatic encephalopathy pathology [7] and self-reported executive dysfunction, depression, apathy and behavioural dysregulation [8]. Despite this, neither study accounted for magnitude, and therefore it is unclear whether HAEs (or SAEs) of 10 or 15 *g* are clinically significant, or that they simply correlate with higher magnitudes that are clinically significant.

**Fig. 2:**
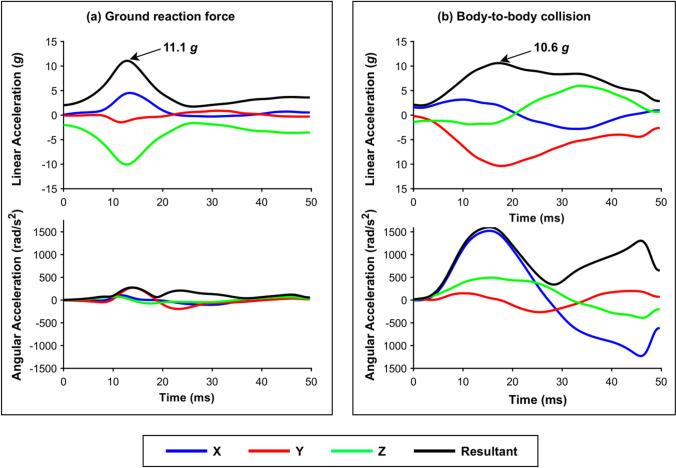
Two sensor acceleration events recorded during **a** a ground reaction force during running [54] and **b** a body-to-body collision [65] in rugby union

Magnitudes of up to 6, 8 and 10 *g* have been recorded during roller coaster rides [53], non-contact events in rugby union [54] and trampolining [55], respectively. Consequently, it has been suggested that HAEs below these magnitudes are unlikely to have a clinical effect. However, thresholds for clinical significance should not solely rely on measures of peak linear acceleration (i.e. “*g*-force”), as variations in angular kinematics and pulse duration may occur even with similar peak linear acceleration values (see Fig. 2). Angular kinematics and pulse duration have been shown to influence brain tissue response in simulations [56–58]; therefore, they should be considered when inferring clinical significance. As a result, the lower boundary of clinically significant HAEs remains unclear.

Future research will pursue a metric that can differentiate clinical significance, for cumulative or acute injury mechanisms. Until such a metric is identified, an appropriate approach to data collection using iMGs may be to optimise data collection to record SAEs across a broad range of magnitudes that may have the potential to be clinically significant.

## Approximation of Head Acceleration Events Using iMGs

The approximation of HAEs is made by recording SAEs, which are short periods of sensor measurements that are processed to approximate head kinematics during an HAE (Fig. 1). An SAE is recorded when inertial sensor measures exceed a pre-determined trigger threshold [1, 18, 23]. The key processing steps for approximating HAEs using SAEs include the filtering of inertial sensor signals to remove electrical noise that does not represent head movement [1, 59]; the transformation of linear kinematics to the head centre of gravity (CoG) to best describe acceleration of the head [1, 60]; and the removal of false-positive SAEs that do not occur during an HAE [1]. False-positive SAEs include those triggered by non-head movement (e.g. biting down on the device or electrical noise), or simply head accelerations that are not caused by HAEs (e.g. voluntary head movements from running). These events are removed by video verification or post-processing algorithms [1, 61, 62]. Typically, post-processing algorithms operate using machine learning to determine which SAEs are true positives and removing those that are not [1]. As a result, an HAE is only approximated by an iMG if both of the following occur: first, the sensor measures must exceed the trigger threshold to record an SAE, and second, the SAE must be retained following post-processing algorithms [1, 61, 62] and/or video verification [1] (i.e. must not be deemed to be a false positive).

## iMG Validity

The technical capability of iMG systems to approximate HAEs has been assessed in validation studies [18–20, 63]. A video analysis of SAEs has been conducted to report positive-predictive and sensitivity values [64]. Positive-predictive values reflect an iMG system’s ability to collect true-positive SAEs without recording false-positive SAEs, while sensitivity values measure the system’s ability of iMGs to collect true-positive SAEs without recording false negatives, respectively. The accuracy of head kinematics is typically assessed in vitro [18–20]. High kinematic accuracy (concordance correlation coefficient values of 0.97–0.99 [18]) and positive-predictive values (positive predictive value of 0.99 in rugby union [65]) have been achieved by iMG systems. Tight coupling with the upper dentition [21] and filtering techniques [59] improve kinematic accuracy, while post-processing algorithms are effective for removing false positives. Reported sensitivity values have been relatively low, with a range from 0.40 to 0.75 between iMG systems following rugby league collisions [18], and 0.80 from head contacts in American Football [66]. This suggests that iMG systems are more likely to suffer from false negatives than false positives.

## False-Negative Mechanisms

**Fig. 3:**
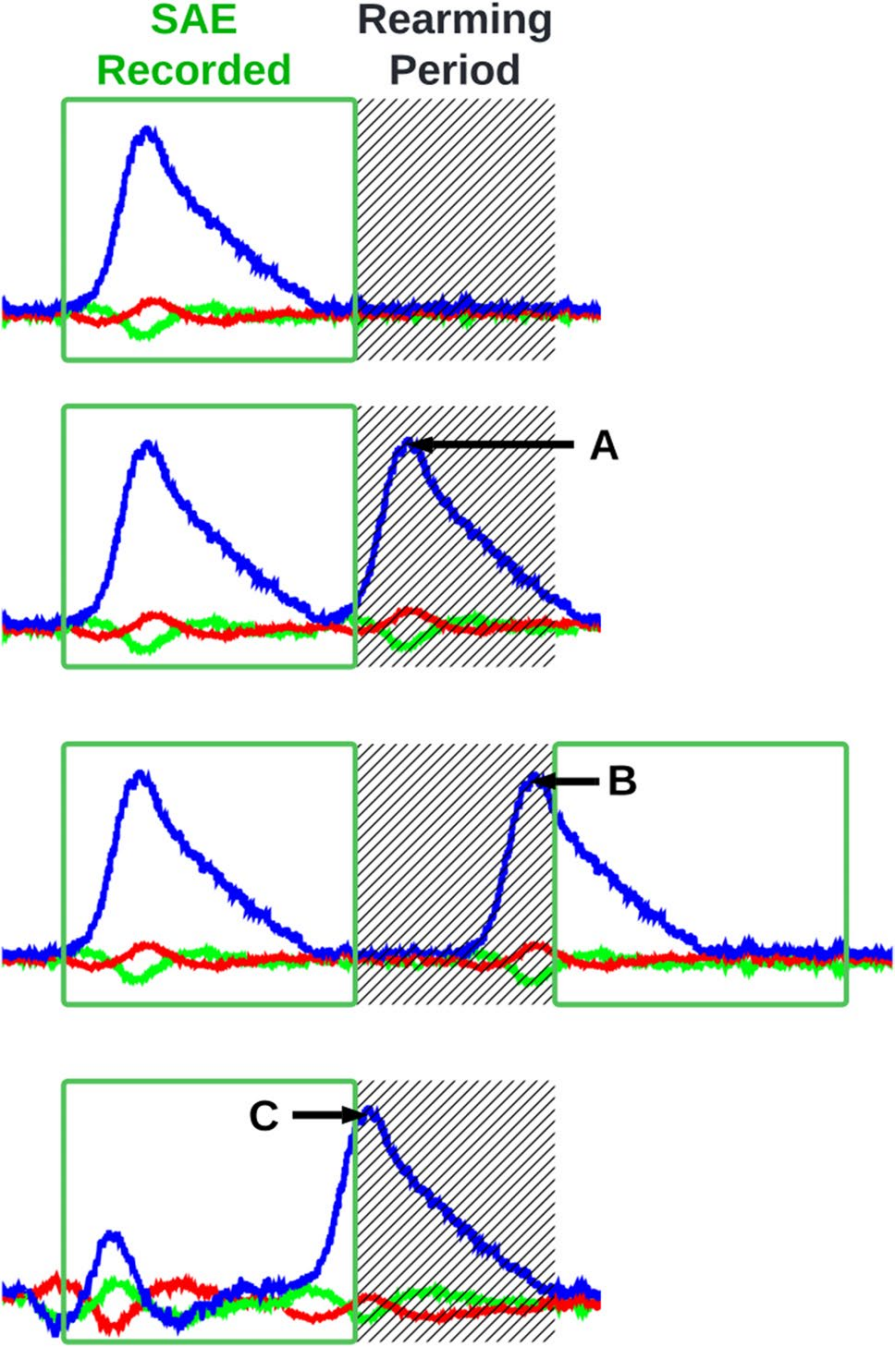
The re-arming period is a short period of time following the collection of a sensor acceleration event (SAE) when the instrumented mouthguard cannot record data. The re-arming period can lead to instrumented mouthguards missing (**A**) or partially missing head accelerations before (**B**) or after (**C**) the SAE

False negatives may occur because of various factors. The misclassification of SAEs by post-processing algorithms can lead to false negatives [1], while the re-arming period may prevent SAEs from being recorded if they occur in quick succession of another SAE. The re-arming period is a brief interval following the recording of an SAE while the event is written to fixed memory; during this period, an iMG is unable to record head kinematics. Figure 3 shows scenarios where the re-arming period may cause false negatives and partially missed SAEs.

In current published research, iMG systems are configured with trigger thresholds [1, 24]. This recording mechanism can contribute to false negatives if an HAE fails to produce sensor measurements exceeding the pre-determined threshold value. The iMG system configured with the lowest trigger threshold in a recent validity study also had the highest sensitivity value [18]; therefore, lowering trigger threshold values should improve false-negative performance. However, it is worth noting that other devices in the study may also be capable of configuring lower trigger thresholds and achieving similar sensitivity values.

## Linear Acceleration Trigger Bias

It is common to assume that HAEs that fail to exceed trigger thresholds are inherently low in magnitude and may be considered as true negatives. However, this assumption is incorrect. Simulations have revealed that HAEs up to 30 *g* in magnitude may fail to exceed a 10 *g* trigger threshold because of the linear acceleration trigger bias [67]. It is crucial that researchers and iMG users recognise how the linear acceleration bias can contribute to false negatives with magnitudes higher than the trigger threshold.

**Fig. 4:**
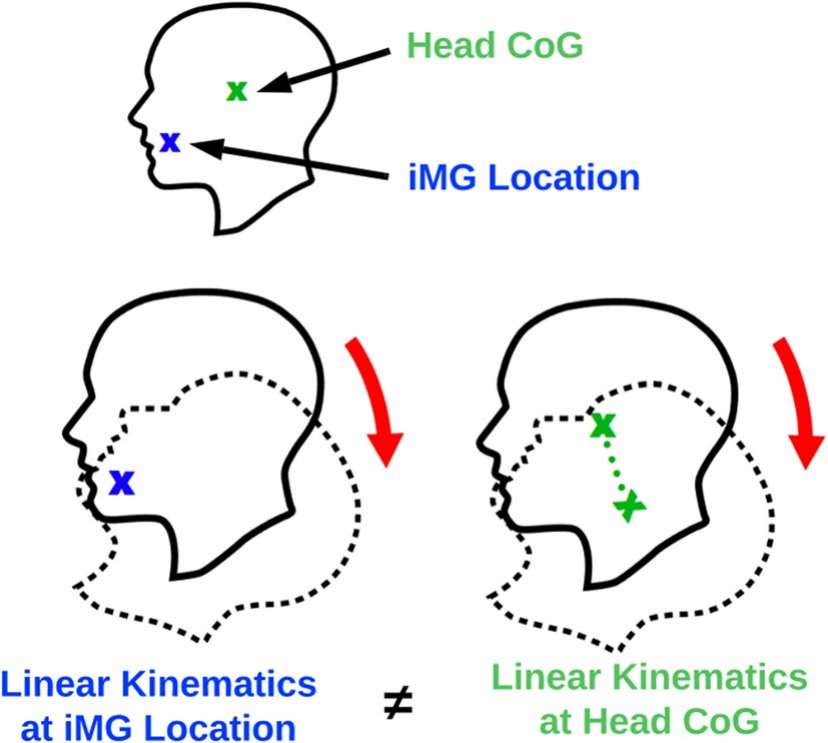
Linear kinematics at the instrumented mouthguard (iMG) location are different to the linear kinematics at the centre of gravity (CoG). In this example, there is no linear acceleration at the iMG location, but there is at the head CoG

The linear acceleration bias [67] occurs because trigger thresholds operate using linear acceleration measured at the iMG location, whereas the linear magnitude of HAEs (and SAEs) is described using resultant values transformed to the head CoG. Linear kinematics are transformed to the head CoG using the relative acceleration equation (Eq. 1). The magnitude of linear acceleration at the iMG location and the head CoG are not always the same [60]. For example, consider an HAE whereby the head merely rotates about the iMG location (Fig. 4); in such a head movement, there would be no linear movement at the iMG location and therefore no linear acceleration. Conversely, the head CoG would move from the start to the end position and therefore experience some degree of linear acceleration. In this way, HAEs can be low in linear acceleration magnitude at the iMG location, but high in magnitude at the head CoG. In some cases, HAEs with high linear acceleration at the head CoG may fail to exceed a linear acceleration trigger threshold at the iMG location, thereby resulting in false negatives.

Simulations of HAEs across various head impact locations, each resulting in a 10 *g* linear acceleration at the head CoG, revealed that only 25% of impact locations exceeded a 10 *g* trigger threshold at the iMG sensor location [67]. The same simulations of 20 *g* HAEs (at the head CoG) resulted in 86% of impact locations exceeding a 10 *g* trigger threshold, while 99.9% of 30 *g* HAEs exceeded 10 *g*. Therefore, false negatives are less likely to occur at higher magnitudes.

1 $$\overrightarrow{{{{a}}}_{h}}=\overrightarrow{{{{a}}}_{m}}+\overrightarrow{{\alpha }}\times {r}_{mh}+\overrightarrow{\omega }\times \left(\overrightarrow{\omega }\times {r}_{mh}\right).$$

The relative acceleration equation, where $\overrightarrow{{{{a}}}_{{{h}}}}$ is the linear acceleration at the head CoG with respect to time, $\overrightarrow{{{{a}}}_{m}}$ is the linear acceleration at the iMG sensor location with respect to time, $\overrightarrow{{\alpha }}$ is the angular acceleration with respect to time, ${r}_{mh}$ is the position vector from iMG sensor location to the head CoG, and $\overrightarrow{\omega }$ is angular velocity with respect to time.

## Current Head Acceleration Exposures are Likely to be Underestimated

In current research, trigger thresholds operate exclusively on linear acceleration values, having been set at 5 [68], 8 [65], 10 [24] and 13 [18] *g*, with 10 *g* being the most common [24]. Similarly, most research papers approximate HAEs by reporting SAEs of 10 *g* and above [24, 69]. Given that prior simulation demonstrates that HAEs as high as 30 *g* may fail to exceed 10 *g* at the iMG sensor location [67] and that a 10 *g* trigger threshold is most common [69], the rate of false negatives caused by the linear acceleration trigger bias is likely to be relatively high in current iMG studies, especially at lower magnitudes. Moreover, head impact locations to the front of the head have been shown to be more common using iMGs in some sports [66, 70], and simulations indicate that these impact locations are more likely to result in false negatives than rear-sided impacts [67]. Therefore, false-negative rates may be even higher in some sports than estimated in previous simulations, which simulated head impacts evenly across head impact locations around the northern hemisphere of the head [67].

The presence of false negatives may lead to an underestimation of HAE exposure, whereas false positives can lead to an overestimation. As video verification and post-processing algorithms virtually eradicate false positives, and current trigger mechanisms result in false negatives, research using iMGs is more likely to underestimate HAE exposures. It is crucial that the interpretation of iMG datasets is made with consideration for the presence of false negatives, particularly at lower magnitudes (< 30 *g*). One approach may be to design research questions to account for limitations of iMG systems. For example, given that false negatives may occur up to 30 *g* when using a 10 *g* trigger threshold [67], future research questions could be designed to focus only on HAEs above 30 *g*, acknowledging that HAE with magnitudes lower than this are harder to detect using iMGs.

## Reducing False Negatives

Various approaches have been recommended for reducing false negatives by mitigating the linear acceleration trigger bias [67]. Angular trigger thresholds have been recommended as an alternative to linear-based triggers because angular kinematics are the same at the sensor location and head CoG under the assumption that the head is a rigid body. However, angular-based sensors can have slower response rates, which may delay triggering and potentially result in more false negatives.

False negatives can be reduced simply by lowering trigger thresholds. This would result in more SAEs being collected and reduce the number of false negatives [67]. However, lowering the trigger threshold would not eradicate the bias and would present additional challenges, including an increased burden on video verification and post-processing algorithms, an increased likelihood of missed HAEs because of the re-arming period, and battery life and storage capacity limitations. An alternative method to avoid false negatives entirely is to continually record and store head kinematics and extract SAEs from a continuous kinematic signal whenever an HAE is identified; however, this approach was ineffective in a recent validation study because of the low sampling rate required to preserve battery life and accommodate storage limitations [18]. Future research should focus on improving iMG design to reduce false negatives.

## Conclusions

With the ability to approximate in vivo head accelerations, iMGs present the opportunity to monitor HAEs during sport. These data can have a multitude of applications across medical, performance and sporting governance settings. To realise this potential, stakeholders (i.e. practitioners, researchers, iMG manufacturers) should consider the conceptual considerations for approximating HAEs in sport using iMGs. For the purposes of this article, the distinction is made between HAEs, the in vivo acceleration event and SAEs, the recorded datum of an iMG for approximating HAEs, to describe how the accuracy of approximation is constrained by technical limitations of iMG systems. Future iMG studies may use the term HAE interchangeably with an SAE; however, they must recognise the technical constraints on iMGs for approximating HAEs outlined in this article.

There is a risk of overestimating HAE exposures if there are a high number of false positives; however, current post-processing algorithms and video verification virtually eradicate these events. Conversely, mitigating false negatives poses a greater challenge, as indicated by relatively lower sensitivity values observed in validations [18, 65]. This suggests that iMGs are more likely to underestimate HAE exposures than to overestimate them. The most pertinent mechanism of false negatives is the linear acceleration trigger bias [67], which can result in HAEs up to 30 *g* failing to exceed a 10 *g* trigger threshold. Consequently, it may be appropriate to focus research questions on magnitudes where iMG systems can sensitively detect HAEs by accounting for the linear acceleration trigger bias. It is crucial that future research mitigates the linear acceleration bias. Until then, the interpretation of existing iMG datasets should be made with consideration for the presence of false negatives, particularly at lower magnitudes (< 30 *g*).

## Declarations

### Availability of Data and Material

Data sharing is not applicable to this article as no datasets were generated or analysed in its preparation.
